# Establishment and Identification of Patient-Derived Xenograft Model for Oral Squamous Cell Carcinoma

**DOI:** 10.1155/2022/3135470

**Published:** 2022-09-05

**Authors:** Fei He, Xiongming Zhou, Gan Huang, Qingkun Jiang, Li Wan, Jiaxuan Qiu

**Affiliations:** Department of Stomatology, The First Affiliated Hospital of Nanchang University, Nanchang 330006, Jiangxi, China

## Abstract

Oral squamous cell carcinoma is the most common head and neck malignancy with high morbidity and mortality. Currently, platinum-based chemotherapy is the conventional chemotherapy regimen for patients with oral squamous cell carcinoma. However, due to the heterogeneity of tumors and individual differences of patients, chemotherapy regimens lacking individualized evaluation of tumor patients are often less effective. Therefore, personalized tumor chemotherapy is one of the effective methods for the future treatment of malignant tumors. The patient-derived xenograft model is a relatively new tumor xenograft model that relies on immunodeficient mice. This model can better maintain various histological characteristics of primary tumor grafts, such as pathological structural features, molecular diversity, and gene expression profiles. Therefore, the patient-derived xenograft model combined with drug screening technology to explore new tumor chemotherapy is the critical research direction for future tumor treatment. This study successfully established the patient-derived xenograft model of oral squamous cell carcinoma. It was verified by hematoxylin-eosin staining and immunohistochemistry that the constructed patient-derived xenograft model retained the pathological and molecular biological characteristics of primary tumors. Our patient-derived xenograft model can be used further to study the oncological characteristics of oral squamous carcinoma and can also be applied to personalize the treatment of oral squamous carcinoma patients, providing a practical resource for screening chemotherapy drugs.

## 1. Introduction

Oral squamous cell carcinoma (OSCC) is the most common head and neck malignant tumor [1]. It is estimated that there are about 500,000 new cases of OSCC (2/3 of them with local infiltration and regional lymph node metastasis) and 350,000 deaths per year worldwide [2, 3]. Although diagnostic methods and clinical treatment techniques have improved dramatically in recent decades, the 5-year survival rate of OSCC patients is still only 40–50% [4, 5]. Chemotherapy resistance is a fundamental reason for their high recurrence and low survival rates [6]. Surgery combined with an extended sequence of preoperative induction chemotherapy is the most effective treatment option for OSCC [7]. However, due to the heterogeneity of tumors and individual differences of patients, the current chemotherapy for solid tumors mainly relies on clinical experience and lacks individualized evaluation and drug guidance for tumor patients, resulting in the poor overall efficacy of chemotherapy [8]. Therefore, accurate selection of effective chemotherapeutic drugs, exploration and discovery of low-toxicity and high-efficiency “individualized” chemotherapy regimens for each tumor and each individual is an effective way to reduce adverse drug reactions and cellular drug resistance and has become an important direction of current individualized chemotherapy research [9].

The development of an individualized chemotherapy regimen requires a biological model that closely mimics OSCC and preserves the primary tumor's stromal heterogeneity, histological characteristics, molecular diversity, and microenvironmental characteristics [10]. A patient-derived xenograft model (PDX model) is a xenograft model in which fresh tumor tissues from patients are directly transplanted onto immunodeficient mice, relying on the growth environment provided by the immunocompromised mice [11]. This model can maintain various histological characteristics of the primary tumor, such as pathological structural features, molecular diversity, and gene expression profile, very well compared to other tumor models [12, 13]. PDX models combined with clinical data, genomic profiles, and pharmacodynamic data can increase drug specificity, be applied to individualized treatment of tumor patients, and improve clinical success rates [14, 15]. It provides a practical R&D resource for preclinical personalized screening assessment of drug efficacy [16].

In this study, we successfully established a PDX model of OSCC that preserved the stromal heterogeneity, histological characteristics, and microenvironment of the primary tumor.

## 2. Materials and Methods

### 2.1. Tumor Samples

The tumor specimens in this study were obtained from patients with OSCC who underwent surgery at the First Affiliated Hospital of Nanchang University from 2018.10 to 2021.10 and were approved by the review committee. The inclusion criteria were as follows:

1. Pathological findings confirmed OSCC
2. Age 18 to 80 years, regardless of gender
3. Tumor site: tongue, gingiva, the floor of the mouth, buccal mucosa, hard palate, and posterior molar area
4. No previous treatment for OSCC
5. Distant metastases were excluded by systemic examination
6. Patients who voluntarily signed the informed consent form

The clinicopathological data of all patients participating in the experiment were collected, including details of patients' age, gender, pathological type, local infiltration, lymph node metastasis, and clinical stage of TNM at the lesion site level.

Tumor specimens were collected surgically for less than 30 min, and the most representative tumor tissues in the tumor foci were selected as far as possible, avoiding the tumor's central liquefied and necrotic part. The tumor tissues were cut into small pieces of about 1 cm × 1 cm × 1 cm, placed in centrifuge tubes containing PBS solution, stored in an ice bath, and sent to the animal laboratory within 1 hour for PDX model construction experiments.

### 2.2. Animals

Balb/c-Nu male nude mice of 6 weeks of age were selected for the experimental animals. The experiments with nude mice were conducted following the guidelines approved by the Experimental Animal Welfare Ethics Committee of the First Affiliated Hospital of Nanchang University. The experimental nude mice were housed in an SPF animal laboratory with a controlled temperature of 22°C∼26°C, relative humidity of 40%∼60%, and a housing density of no more than five animals/cage, and the cages, drinking water, bedding, and feed were sterilized. All animal experiments were conducted in specific pathogen-free (SPF) animal laboratory biosafety cabinets.

### 2.3. PDX Modeling

The surgically resected tumor samples were further processed to remove evident necrosis, liquefaction, and other tissues without tumor activity; the treated tumor tissue was stained with trypan blue solution for about 3 minutes, and the tumor tissue activity was detected. If the staining rate was lower than 50%, the tumor sample has good activity and can be used for subsequent experiments. Tumor samples were further trimmed into 2 mm × 2 mm × 2 mm tissue blocks with a scalpel blade and placed in Matrigel containing antibiotics. In an ultra-clean biological safety cabinet, the nude mice were anesthetized with isoflurane and then fixed, and the skin of the right scapular region of the nude mice was disinfected three times with iodophor. The treated tumor tissue is filled into the inoculation trocar with a microscopic instrument so that the skin is under a particular tension state, the inoculation needle is pierced into the skin, and the tumor tissue is slowly pushed out after the needle is inserted into the lower edge of the scapula. After withdrawing the needle, we re-sterilize the needle insertion site. After the inoculation was completed, the number was recorded on the same side of the ear of the nude mouse with an ear pin. The tumor volume was calculated by regularly measuring the length and short diameter of the tumor [17]. When the tumor volume reached 1000–2000 mm^3^, it indicated that the PDX model of the P0 generation was successfully constructed. Under sterile conditions, the nude mice were anesthetized, the tumors were removed entirely, and the tumors were processed according to the construction method of the primary model and then transplanted into the next generation of nude mice to form the P1 generation PDX model. The original measurement method and construction method were still passed on in sequence until it was passed on to the P3 generation PDX model. After the P3 generation tumor tissue was peeled off, a part of the tissue was tested for pathology and molecular biology, the remaining tumor tissue was subjected to programmed cooling, and then the samples were placed at -80 °C for cryopreservation and reserved for subsequent experiments [18].

### 2.4. Histology and Immunohistochemistry

The tumor tissue of the PDX model passed to the P3 generation was selected for pathological analysis. The surgically peeled transplanted tumor tissue samples were fixed with 4% formaldehyde, embedded in paraffin, cut into 2- to 3-*μ*m sections, and stained using hematoxylin-eosin. Sections from tissue blocks of the tumors studied were immunohistochemically stained with the following antibodies: P53 (clone 6C4B6, dilution 1:200, Proteintech, USA). Images were captured using a microscope, and P53 expression was evaluated by counting the number of positive cells under a light microscope at a magnification of ×400. Data are presented as the percentage of positive cells.

### 2.5. Statistical Analysis

Statistical analyses were performed using GraphPad Prism. The data are expressed as the mean ± SEM unless indicated otherwise. Unpaired Student's *t*-test was used to determine statistically significant differences. *P* < 0.05 was considered significant at the 95% confidence level.

## 3. Results and Discussion

### 3.1. Successful Establishment of the OSCC PDX Model

**Figure 1:**
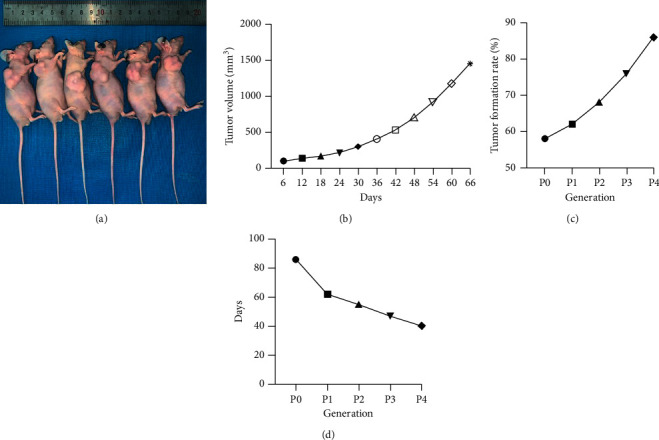
Data related to the construction of PDX model for oral squamous carcinoma. (a) The constructed PDX model for oral squamous carcinoma. (b) The tumor growth curve of OSCC PDX model. (c) Tumor formation rate of the OSCC PDX model at different generations. (d) Tumor formation time of the OSCC PDX model at different generations.

We successfully established 12 PDX models of OSCC (Table 1, Figure 1(a)), and they were passed from P0–P3 generations. During the observation of PDX models, we found that tumor volume growth in PDX models was slow in the early stage of tumor formation. However, the tumor volume growth rate accelerated significantly as time progressed, which was similar to the growth course of solid tumors, indicating that PDX models retained the biological characteristics of tumors (Figure 1(b)). We recorded the establishment process of the PDX model for these 12 cases of OSCC in detail and found that the tumor formation rate of the PDX model increased gradually with the increase of the number of generations, and the tumor formation time decreased continuously with the increase of the number of generations (Figures 1(c) and 1(d)). Moreover, we observed that the tumorigenesis time and tumorigenicity rate of PDX models of OSCC varied considerably among individuals, and we believe that the tumorigenicity time of PDX models of OSCC is related to the tumorigenicity rate and the pathological and biological characteristics of the original tissues, such as the depth of infiltration, the presence or absence of lymph node metastasis, and the pathological grading.

### 3.2. The PDX Model Preserves the Pathological Features of the Primary Tumor

**Figure 2:**
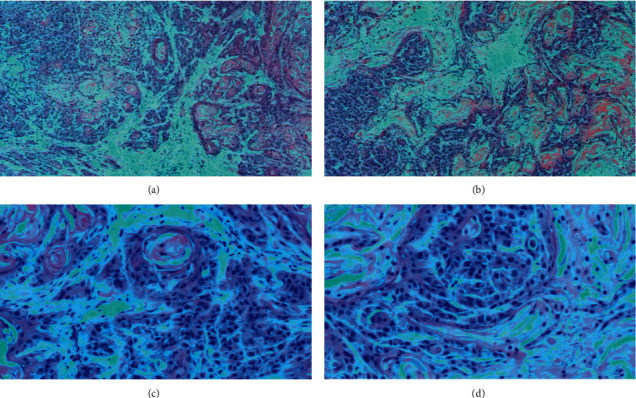
Hematoxylin-eosin staining. (a, c) Primary tumor tissue of the OSCC PDX model (hematoxylin-eosin, 40×, 200×); (b, d) tumor tissue of PDX model of OSCC (hematoxylin-eosin, 40×, 200×).

We selected the P3-generation PDX model and compared the histomorphological characteristics of the original tumor tissue and the PDX model tumor tissue by HE staining. We found that the histomorphology of the two was highly similar, including pathological grading, nuclear division, and offset (Figure 2). This indicates that the PDX model tumor tissue we constructed has high fidelity in pathological histomorphology.

### 3.3. PDX Model Preserves the Molecular Phenotype of Primary Tumor Tissue

**Figure 3:**
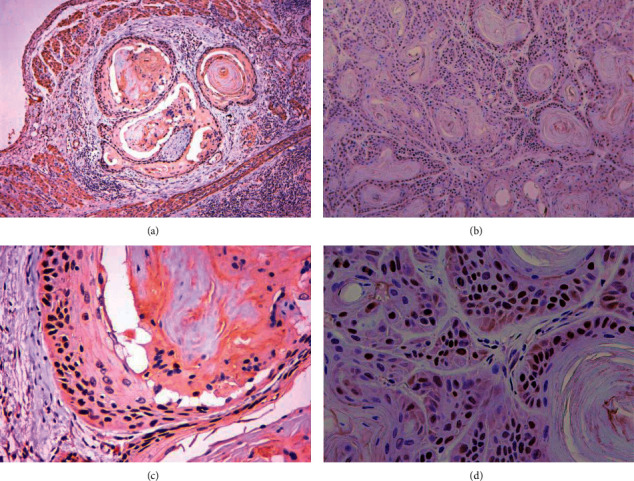
Immunohistochemistry. (a, c) P53 expression in primary tumors of the PDX model of OSCC (IHC, 40×, 200×); (b, d) P53 expression in PDX model tumors of OSCC (IHC, 40×, 200×).

To confirm that the PDX model of OSCC preserved the molecular tissue phenotype of the primary tumor tissue, we also performed immunohistochemistry. Tumor tissues from the P3 generation PDX model were also selected to compare the expression of cell cycle protein P53 with that of the primary tumor tissues. P53 is an important tumor suppressor gene and the most studied oncogene-related gene, which is involved in the regulation of the cell cycle and apoptosis. Several studies have shown that the positive expression rate of P53 in patients with OSCC is about 70%. We can see from the immunohistochemical results (Figure 3) that the tumor tissues of our constructed PDX model of OSCC and the P53 of the original tumor are identical in expression, which indicates that the PDX model retains the molecular biological characteristics of the original tumor tissue and preserves the aggressive and growth characteristics of the original tumor tissue.

## 4. Discussion

For early-stage OSCC, the prognosis is reasonable after surgery and radiotherapy, but for the middle and late stage, especially for incomplete resection of advanced OSCC, surgery alone with postoperative radiotherapy will not have an excellent prognosis [19]. Studies have shown that effective preoperative induction chemotherapy can significantly improve progression-free survival and overall survival of patients with OSCC [20]. Chemotherapy for OSCC is mainly based on the combination of platinum-based first-line chemotherapy drugs given by NCCN [21]. In contrast, the PDX model for OSCC combined with drug screening technology can screen tumor patients' most appropriate chemotherapeutic agents and form a personalized treatment plan. The PDX model has a tremendous advantage over other tumor models in retaining tumor heterogeneity [22]. In histopathology, the PDX model retains the original tumor structure and stromal components, which can reflect the relationship between tumor cells and their microenvironment and stimulate the growth, metastasis, and angiogenesis of human tumor tissue [15, 23, 24]. At the cellular level, PDX models can accurately reflect original cancer's phenotypic and molecular characteristics. These advantages make PDX models promising for drug efficacy studies and clinical prognosis prediction [25, 26]. PDX models have been used mainly in lung, rectal, pancreatic, glioblastoma, and other cancers and are less studied in oral squamous carcinoma. [27, 28, 29, 30]. Although PDX models are widely used in cancer treatment, there are many limitations and shortcomings in the clinical application of PDX models. The modeling time period of PDX models is long, the success rate of modeling is unstable and expensive, the difficulty of modeling different types and stages of cancer varies, and the modeling speed of different modeling approaches also varies [31]. Even now, various immunodeficient mice can shorten the modeling time [32], but in general, cancer progresses faster than the speed of PDX modeling, which leads to PDX models for patients not directly benefit patients, but more for basic research of cancer treatment.

Moreover, the frequency of genome-wide allelic variants during successive passages in PDX tumors suggests that clonal selection occurs more frequently in the initial transplantation step than in the passaged amplification step, while specific clonal selection varies across tumor samples of the same tumor type [33]. In addition, the PDX model is constructed using immunodeficient mice, which do not possess immune cells and immune systems and cannot be used to detect immune responses, so it is challenging to combine cutting-edge cancer immunotherapy with the PDX model. However, some scholars have proposed to inject human hematopoietic stem cells or peripheral blood mononuclear cells into mice to build a humanized PDX mouse model, which can mimic the human immune system to a certain extent, thus enhancing the research value and application prospects of PDX models [34, 35, 36].

We compared the primary patients' clinical characteristics in establishing the PDX model of OSCC. We found that tumor tissues with more advanced pathological stage, higher tumor infiltration, and lymph node metastasis were more likely to become tumorigenic in the PDX model [37], which we considered to be caused by the different value-added activities of tumor cells, which is an essential guideline for the establishment of the PDX model in the future. At the same time, we combined a tumor drug screening technology to explore the personalized treatment of OSCC, and we have achieved good experimental results [38].

## 5. Conclusions

In this study, the PDX model of OSCC was successfully established, and it was identified that the PDX model preserved the pathological structure and molecular biological characteristics of the original tumor tissue, which can be used as a preclinical model to study the treatment of OSCC.

## Figures and Tables

**Table 1:** Clinicopathological features of primary tumor patients with the PDX model of OSCC.

Age	Gender	Location	Pathology	TNM stage
42	M	Left lingual edge	Highly differentiated squamous cell carcinoma.	T2N0M0
54	M	Right lingual edge	Highly differentiated squamous cell carcinoma.	T2N0M0
61	F	Right cheek	Keratinizing squamous cell carcinoma with invasion of transverse muscle tissue.	T3N0M0
54	M	Lower left gum	Moderately differentiated squamous cell carcinoma, invading bone tissue.	T4aN0M0
74	M	Right lingual edge	Keratinizing squamous cell carcinoma with invasion of submucosal transverse muscle tissue and salivary gland tissue.	T4aN0M0
44	M	Right lingual edge	Squamous cell carcinoma with invasion of the transverse muscle and metastasis to one lymph node.	T3N1M0
48	M	Right lingual edge	Squamous cell carcinoma, invading the transverse muscle and salivary gland tissue and a lymph node metastasis.	T4aN1M0
74	F	Left lingual edge	Squamous cell carcinoma with two lymph node cancer metastases visible in the left neck.	T3N2bM0
88	M	Lower left gum	Highly differentiated squamous cell carcinoma invading transverse muscle and bone tissue, two lymph node carcinoma metastases visible in the left neck.	T4aN2aM0
44	F	Right lingual edge	Keratinizing squamous cell carcinoma with invasion of transverse muscle tissue and two lymph node cancer metastases in the right neck.	T4aN2bM0
68	M	Left lingual edge	Squamous cell carcinoma with invasion of transverse muscle tissue. One lymph node metastasis was seen in the left neck. Two lymph node carcinoma metastases were seen in the right neck.	T4aN2cM0
42	M	Left lingual edge	Squamous cell carcinoma involves transverse muscle tissue invading the nerve. Four lymph node carcinoma metastases were seen in the left neck and three lymph node carcinoma metastases were seen in the right submandibular.	T4bN2cM0

## Data Availability

The data used to support the findings of this study are available from the corresponding author upon request.
